# Typical Misconceptions Pertaining to Facepiece Masks against Viral Aerosols

**DOI:** 10.3390/toxics12060443

**Published:** 2024-06-20

**Authors:** Byung Uk Lee

**Affiliations:** Bioaerosol Laboratory, Konkuk University, 120 Neungdong-ro, Gwangjin-gu, Seoul 05029, Republic of Korea; leebu@konkuk.ac.kr; Tel.: +82-2-450-4091

**Keywords:** aerosol, facepiece mask, respirator, viral aerosol, infectious diseases

## 1. Filtration Principles of Facepiece Masks against Viral Aerosols

Since the early stages of the COVID-19 pandemic, there have been debates regarding the transmission modes of contagious viruses, including the influenza virus and severe acute respiratory syndrome coronavirus 2 (SARS-CoV-2), along with its variants [1,2,3]. This debate has continued during the outbreaks of human mpox (formally known as monkeypox) virus [4,5,6].

For infectious diseases, in addition to direct physical contact with the viral lesions of patients or the fluids contained within them during intimate contact, the following three modes of transmission of contagious viruses have been widely discussed: fomite, droplet, and aerosol transmission. Fomite transmission refers to the spread of the virus via direct contact of potential hosts with inanimate object surfaces containing the virus. Droplet and aerosol transmission refer to viral infections via exposure of the respiratory tracts of potential hosts to droplets (i.e., earthbound respiratory particles) that carry viruses and viral aerosols (i.e., airborne viral particles), respectively.

In many countries, including the United States and South Korea, the unsolved conclusions regarding this transmission issue have caused conflicts when developing preventive protocols such as the public mandatory requirement of wearing facepiece masks; consequently, the phenomenon might have enabled the extensive spread of these viruses during outbreaks. Some countries, such as the United States, did not initially recommend that the general public wear facepiece masks during the early stages of the pandemic; however, other countries, such as South Korea, did advise their citizens to wear facepiece masks.

As evidence of aerosol transmission of contagious viruses such as SARS-CoV-2, influenza, and human mpox has accumulated [1,2,3,4,5,6,7], air filtration as a control method against viral aerosols has received considerable attention.

However, many clinicians misunderstand an air filtration principle and its underlying aerosol dynamics. They believe that facepiece respirators, which typically consist of micrometer-scale filter fibers, cannot capture 100 nm sized viral aerosols (after desiccation of moisture from the respiratory tract) because of the larger size of the filter mesh. Therefore, they conclude that it is impossible to control viral aerosols such as SARS-CoV-2 aerosols, using typical air filters in facepiece masks. However, this common belief is incorrect.

Air filters used against aerosol particles do not work as sieves. With a sieve, only particles smaller than the size of the mesh (hole) of the sieve can pass through, and many larger particles are blocked by the mesh. However, the air filters in facepiece masks work differently. Aerosol filtration is based on aerosol dynamics and fluid dynamics. Four mechanisms support aerosol filtration: impaction, interception, diffusion, and electrophoresis [8,9].

The inertia of aerosol particles is larger than the inertia of air molecules. Therefore, these inertia characteristics induce collision of aerosol particles onto blocking filter fibers when particle-carrying air flows change directions inside filters. This is called impaction. The effect of the impaction principle intensifies as aerosol particles increase in size. For instance, particles sized 10 µm are strongly influenced by the impaction principle, whereas particles measuring 0.1 µm are minimally affected.

When an aerosol particle touches the surface of filter fibers during passing, it sometimes sticks to the surface, a phenomenon called interception. Moreover, tiny aerosol particles spread to filter fibers as they pass through a filter by a process called diffusion. The diffusion effect strengthens as aerosol particles decrease in size, exhibiting the opposite trend of the impaction principle. For example, the diffusion principle significantly influences 0.1 µm particles; however, 10 µm particles are barely affected.

If the filter fibers are electrically charged, they can attract aerosol particles to their surface, which is called electrophoresis. These four principles enable air filters to capture nanometer-size aerosol particles with hundreds of micrometer-size filter fibers. Therefore, facepiece masks can capture many viral aerosols, and the minimum efficiency of ordinary masks occurs against 0.3 µm size particles.

In special masks, various materials including activated carbons are inserted to reduce the passage of airborne chemicals. Furthermore, improved methods using new types of fibers, thermal energy, photocatalysts, and ions have been tried to supplement facepiece masks [10].

These analyses of filtration principles provide foundational insights for in-depth discussions on facepiece masks and viral aerosols, helping to dispel numerous misconceptions pertaining to the filtration principles of facepiece masks.

## 2. Benefits of Using Facepiece Masks

Facepiece masks can significantly reduce exposure to external aerosol particles in the human respiratory tract. This reduction is evident when comparing concentrations of aerosol particles inside and outside the human mouth while wearing a facepiece mask [8,9,10,11]. The rate of reduction in aerosol particle concentration due to wearing facepiece masks approaches >90% with a proper fit between the mask and the human face [11]. Thus, it is clear that wearing masks decreases the risk of respiratory exposure to external hazardous aerosol pollutants, including airborne pathogens. Besides this direct measurement studies, a real-world statistical study in Massachusetts schools in the Unites States found that removing mask mandates was associated with an increase of 44.9 COVID-19 infection cases per 1000 students and staff in schools in Massachusetts [12]. Therefore, facepiece masks can be considered as efficient tools to decrease the exposure risk of wearers to external pathogenic particles. In addition, it was reported that the widespread use of masks at the community level had a positive impact on mental health during the COVID-19 pandemic by reducing anxiety, depression, and stress [13].

## 3. Disadvantages of Using Facepiece Masks

Many facepiece masks have been manufactured by layering filters with bonding boundaries. Users of facepiece masks can be exposed to volatile chemical materials which were used in these processes [14]. Facepiece masks can also inhibit respiratory activities, such as inhalation and exhalation, by obstructing airflow through the mouth and nose. Although it has been reported that wearing a surgical mask does not significantly impair respiration in tested humans [15], concerns about the effects of prolonged mask use on respiratory health of wearers still remain. It was reported that extended wearing of masks has been linked to headaches, rashes, and skin irritation [16]. For individuals who wear glasses, masks can cause the lenses to fog up in cold weather, leading to visibility issues. Therefore, it may cause inconvenience for glasses users.

Furthermore, facepiece masks cover human faces, and therefore, it is difficult to recognize the emotions of persons who are wearing masks. Therefore, it can be said that the wearing of masks can obscure facial expressions, thereby reducing the efficiency of communication [16,17]. This may also be connected to mental health among people.

In addition to the above discussions, with the use of facepiece masks worldwide to prevent the spread of airborne coronavirus particles during the COVID-19 pandemic, microbial contamination of masks has received much attention. To manufacture microbial-contamination free masks with air filters, several methods, including the coating of facepiece masks with toxic materials as an antimicrobial agent, have been studied for years [10]. Generally, if a substance can inactivate microorganisms through toxic actions, it may also have the potential to induce adverse effects on surrounding eukaryotes, including humans. Studies on antimicrobial coatings containing toxic chemicals on products that come into contact with the human skin such as facepiece masks should be balanced with considering the potential adverse effects of such chemicals. Disposable masks are recommended for temporary use for several hours before being discarded. Coating single-use masks with toxic materials may have few benefits and can cause serious adverse effects in humans and on the surrounding environment. Hence, fundamental discussions are needed for studies on antimicrobial materials for coating facepiece masks.

## 4. Summary

Overall, incorrect policies against contagious diseases have existed worldwide owing to the misunderstanding of aerosol dynamics. An understanding of aerosol dynamics is necessary to scientifically address the spread of contagious diseases via aerosols. Furthermore, there are many ongoing debates over the benefits and disadvantages of wearing facepiece masks [18]. In the short term, the benefits of wearing facepiece masks are evident; however, in the long term, facepiece masks have several side effects which cannot be overlooked. During any pandemic, policies regarding mandatory facepiece mask-wearing must be carefully evaluated to balance these benefits and drawbacks.

## Data Availability

The data presented in this study are available on request from the author.
